# Clinical Characteristics and Reasons for Differences in Duration From Symptom Onset to Release From Quarantine Among Patients With COVID-19 in Liaocheng, China

**DOI:** 10.3389/fmed.2020.00210

**Published:** 2020-05-12

**Authors:** Suochen Tian, Zhenqin Chang, Yunxia Wang, Min Wu, Wenming Zhang, Guijie Zhou, Xiuli Zou, Hui Tian, Tingfang Xiao, Junmin Xing, Juan Chen, Jian Han, Kang Ning, Tiejun Wu

**Affiliations:** ^1^Intensive Care Unit, Liaocheng People's Hospital, Liaocheng, China; ^2^Department of Hemodialysis, Liaocheng People's Hospital, Liaocheng, China; ^3^Intensive Care Unit, Liaocheng Infectious Diseases Hospital, Liaocheng, China; ^4^Department of Nosocomial Infection, Liaocheng People's Hospital, Liaocheng, China; ^5^Department of Respiratory Medicine, Liaocheng People's Hospital, Liaocheng, China; ^6^Department of Pulmonology, The Affiliated Hospital of Shandong University of Traditional Chinese Medicine, Jinan, China; ^7^Department of Respiratory Medicine, The First Affiliated Hospital of Shandong First Medical University, Jinan, China

**Keywords:** COVID-19 patients, quarantine, epidemiologic characteristic, clinical characteristic, granulocyte count

## Abstract

**Objective:** This study aimed to identify additional characteristics and features of coronavirus disease (COVID-19) by assessing the clinical courses among COVID-19 patients in a region outside Hubei province.

**Methods:** We analyzed retrospective data regarding general characteristics, epidemiologic history, underlying chronic diseases, clinical symptoms and complications, chest computed tomography findings, biochemical monitoring, disease severity, treatments, and outcomes among 37 adult patients with COVID-19. According to the duration from symptom onset to release from quarantine, the patients were divided into the ≤20 and >20-day groups, and the similarities and differences between them were compared.

**Results:** Among the 37 patients, five had mild disease, 30 had moderate disease, one had severe disease, and one was critically ill. All of the patients were released from quarantine, and no mortality was observed. The average duration from symptom onset to release from quarantine was 20.2 ± 6.6 days. The average duration from symptom onset to hospitalization was 4.1 ± 3.7 days, and the patients were hospitalized for an average of 16.1 ± 6.2 days. The average age was 44.3 ± 1.67 years, and 78.4% of cases were caused by exposure to a patient with confirmed disease or the workplace of a patient with confirmed disease. The main symptoms were cough (67.6%), fever (62.2%), shortness of breath (32.4%), fatigue (24.3%), sore throat (21.6%), vomiting, and diarrhea (21.6%). White blood cell count was decreased in 27.0% of patients, and lymphocyte count was decreased in 62.2% of the patients, among whom 43.5% patients had counts of ≤0.6 × 10^9^/L. On admission, 86.5% of patients showed pneumonia in chest CT scans, including some asymptomatic patients, while 68.8% of patients showed bilateral infiltration. In the >20-day group, the average age was 49.9 ± 1.38 years, and the average duration from symptom onset to hospitalization was 5.5 ± 3.9 days. Compared with the ≤20-day group, patients in the >20-day group were older and the duration was longer (*P* < 0.05). All of the seven asymptomatic patients belonged to the ≤20-day group. When the 37 patients were released from quarantine, the white blood cell count of 16.2% of the patients was <4.0 × 10^9^/L, the lymphocyte count of 59.5% of the patients was <1.1 × 10^9^/L, and the absolute counts of white blood cells and lymphocytes were 5.02 ± 1.34 × 10^9^/L and 1.03 ± 0.34 × 10^9^/L, respectively, compared with those recorded on admission (*P* > 0.05).

**Conclusion:** The majority of COVID-19 cases in the study area were mild and moderate, with good clinical outcomes. There were some special characteristics in the clinical course. The reasons for differences in the duration from symptom onset to release from quarantine were complex. There was no significant change in the number of granulocytes at the time of release from quarantine compared to that at the time of admission.

## Background

Previous articles have described the clinical characteristics and outcomes of coronavirus disease (COVID-19) (1–6). These mainly reported on early cases diagnosed in Hubei province, particularly in Wuhan. The limitations imposed by non-optimal medical conditions at that time had some impact on the outcomes and treatment of COVID-19. Differences have been noted in the clinical characteristics and outcomes of patients diagnosed inside and outside Hubei province (1). One article reported on the early clinical characteristics of 13 COVID-19 patients outside Hubei province; however, the number of patients was small, and the article only described the early clinical characteristics (7). Liaocheng city, in the middle east region of China, is a prefecture-level city located in Shandong province with a population of more than 6 million. As a region outside Hubei province, what are the similarities and differences between the characteristics of the cases diagnosed here and those diagnosed in Hubei province and even other countries and regions? Also, are there any special characteristics of patients who cannot be released from quarantine for a long period? These are a few of the questions that need to be answered. This study thus aimed to identify additional characteristics and features of COVID-19 by assessing the clinical courses of COVID-19 patients in a region outside Hubei province.

## Methods

Patient diagnosis, release from quarantine, and disease severity among all cases were determined according to the “Protocol for the Diagnosis and Treatment of Novel Coronavirus Pneumonia” issued by the National Health Commission of the People's Republic of China and the National Administration of Traditional Chinese Medicine (8, 9). A confirmed case was defined by a positive result to real-time reverse-transcription polymerase chain reaction (RT-PCR) assay of nasal and pharyngeal swab specimens (2).

The criteria for release from quarantine for all cases were as recommended in the above protocol, starting with the following three: (1) body temperature returns to normal for more than 3 days, (2) respiratory symptoms improve significantly, and (3) pulmonary imaging shows significant absorption of acute exudative lesions. Based on these criteria, quarantined persons could be released if strictly negative nucleic acid test results were obtained after 5 days in the hospital and in tests performed every 2 days. Individuals for whom nucleic acid tests yielded negative results when instead tested every 24 h could be released from quarantine if three consecutive test results are negative. During the dynamic test, if cases for whom nucleic acid test results were negative showed positive results, the above steps were restarted. Some patients were kept in the hospital for 14 days after they were released from quarantine.

Severe cases were identified in accordance with the respiratory criteria, excluding those who did not meet the respiratory criteria and required intensive care (10).

One of the 38 patients, a 5-month-old child identified by screening and released from quarantine after 9 days in hospital, was excluded from the analysis. The other 37 patients were all adults. Regarding the incubation period and considering the characteristics of the patients in this study, it was difficult to tell the precise time of first infection with SARS-CoV-2; therefore, this was not discussed.

The present study retrospectively analyzed the general characteristics, epidemiological history, chronic underlying diseases, clinical symptoms, complications, chest computed tomography (CT) findings, biochemical features, disease severity, treatment plans, and outcomes of 37 patients. The results of examinations were reported at study time nodes of ±24 h. In addition, these patients were divided into the ≤20-day group and >20-day group according to the duration of release from quarantine. We compared the similarities and differences between the two groups in the clinical process to identify relevant factors among patients who continued to test positive for nucleic acid.

## Statistical Analysis

Continuous variables are expressed as means and standard deviations or as medians and interquartile ranges (IQRs), as appropriate. Categorical variables are summarized as counts and percentages in each category. Continuous variables were analyzed using the *t*-test or Wilcoxon rank-sum test, as appropriate. Categorical variables were analyzed using Fisher's exact test. Rank classification of variables was performed using the Wilcoxon rank-sum test. All statistical analyses were performed using SPSS version 23.0 (IBM Corp., Armonk, NY, USA).

## Results

The duration from symptom onset to admission ranged from 1 to 10 days among the 37 patients with confirmed disease. The shortest length of stay was 7 days, and the longest was 32 days. The shortest duration from symptom onset to release from quarantine was 8 days, and the longest was 34 days. The duration from symptom onset to release from quarantine was 29 days for one patient with severe disease and 11 days for one critically ill patient.

According to the pneumonia severity index (PSI) on admission, 89.2% (17 + 16/37) of patients were classified as at the low risk grades I and II. Regarding epidemiological history, all of the six patients initially diagnosed had a history of sojourn in Wuhan, and patients diagnosed subsequently had mainly been in contact with the confirmed cases or their workplaces. A high proportion of patients had symptoms on admission, including cough, fever, shortness of breath, fatigue, sore throat, vomiting, or diarrhea, and 87.0% of the patients had a low fever. No abnormalities in platelets and levels of creatine kinase or creatinine were observed on routine blood biochemistry tests performed on admission (Table 1). During treatment, two patients had acute respiratory distress syndrome (ARDS) (mild, *n* = 1; moderate, *n* = 1) (11) and received high-flow nasal cannula (HFNC) oxygen therapy without non-invasive or invasive mechanical ventilation. Critically ill patients with moderate ARDS underwent plasma exchange. No patient experienced serious complications such as shock, kidney injury, pulmonary embolism, or diffuse intravascular coagulation. Regarding treatment, one critically ill patient received an antifungal drug, and all patients received two or more antiviral drugs. The order of the rates of application of other therapeutic measures, descending, was as follows: thymosin, oxygen therapy, albumin, hormone, and immunoglobulin. A 100 percent of patients received traditional Chinese medicine (TCM), including Chinese medicine preparations, acupuncture, and moxibustion (Table 2).

**Table 1:** Clinical characteristics of the study patients on admission.

**Characteristics**	**All patients (*n* = 37)**	**Duration from onset to release from quarantine**		
		**≤20-day (*n* = 19)**	**>20-day (*n* = 18)**	***P*-value**
Age (yrs.)	44.3 ± 1.67	39.1 ± 1.79	49.9 ± 1.38	0.047
**Gender**				
Male (%)	17/37 (45.9)	9/19 (47.4)	8/18 (44.4)	0.121
Female (%)	20/37 (54.1)	10/19 (52.6)	10/18 (55.6)	
Duration from onset to hospitalization (d)	3 (5.5)	2 (4)	6 (5.25)	0.016
PSI	45 (105)	51 (53.5)	44.5 (55.75)	0.932
I (%)	17/37 (45.9)	8/19 (42.1)	9/18 (50.0)	
II ≤ 70 (%)	16/37 (43.2)	10/19 (52.6)	6/18 (33.3)	
III 71–90 (%)	3/37 (8.1)	1/19 (5.3)	2/18 (11.1)	
IV 91–130 (%)	1/37 (2.7)	0/19 (0)	1/18 (5.6)	
V >130 (%)	0/37 (0)	0/19 (0)	0/18 (0)	
**Underlying disease**				
Any (%)	8/37 (21.6)	4/19 (21.1)	4/18 (22.2)	1
Hypertension (%)	3/37 (8.1)	1/19 (5.3)	2/18 (11.1)	0.604
Coronary heart disease (%)	3/37 (8.1)	1/19 (5.3)	2/18 (11.1)	0.604
Diabetes (%)	2/37 (5.4)	1/19 (5.3)	1/18 (5.6)	1
Pulmonary Interstitial fibrosis (%)	1/37 (2.7)	0/19 (0)	1/18 (5.6)	0.486
Cirrhosis, liver cancer (%)	1/37 (2.7)	0/19 (0)	1/18 (5.6)	0.486
**Epidemiological history**				
Contact with wild (%)	0/37 (0)	–	–	
Wuhan sojourn (%)	6/37 (16.2)	3/19 (15.8)	3/18 (16.7)	1
Contact with a diagnosed patient or workplace (%)	29/37 (78.4)	15/19 (78.9)	14/18 (77.7)	1
Not clear (%)	2/37 (5.4)	1/19 (5.3)	1/18 (5.6)	1
**Symptoms**				
Asymptomatic (%)	7/37 (18.9)	7/19 (36.8)	0/18 (0)	0.008
Fever (%)	23/37(62.2)	8/19 (42.1)	15/18 (83.3)	1
≤38.0°C (%)	20 (87.0)	7 (87.5)	13 (86.7)	1
>38.0°C (%)	3 (13.0)	1 (12.5)	2 (13.3)	
Chills (%)	1/37 (2.7)	0/19 (0)	1/18 (5.6)	0.486
Fatigue (%)	9/37 (24.3)	1/19 (5.3)	8/18 (44.4)	0.008
Headache (%)	3/37 (8.1)	0/19 (0)	3/18 (16.7)	0.105
Nasal congestion (%)	3/37 (8.1)	2/19 (10.5)	1/18 (5.6)	1
Sore throat (%)	8/37 (21.6)	0/19 (0)	8/18 (44.4)	0.001
Cough (%)	25/37(67.6)	11/19 (57.9)	14/18 (77.8)	0.295
Hemoptysis (%)	1/37 (2.7)	0/19 (0)	1/18 (5.6)	0.486
Shortness of breath (%)	12/37(32.4)	3/19 (15.8)	9/18 (50.0)	0.035
Vomiting or diarrhea (%)	8/37 (21.6)	5/19 (26.3)	3/18 (16.7)	0.693
Pain in a muscle or joint (%)	2/37 (5.4)	1/19 (5.3)	1/18 (5.6)	1
**Laboratory findings**				
WBC <4.0 × 10^9^/L (%)	10/37 (27.0)	4/19 (21.1)	6/18 (33.3)	1
L <1.1 × 10^9^/L (%)	23/37 (62.2)	12/19 (63.2)	11/18 (61.1)	0.366
L ≤ 0.6 × 10^9^/L (%)	10/23 (43.5)	6/12 (50.0)	4/11 (36.4)	0.68
SAA >10 mg/L (%)	21/37 (56.8)	10/19 (52.6)	11/18 (61.1)	0.743
CRP >5 mg/L (%)	19/37 (51.4)	7/19 (36.8)	12/18 (66.7)	0.103
PCT >0.5 ng/ml (%)	1/37 (2.7)	0/19 (0)	1/18 (5.6)	0.486
LDH >250 U/L (%)	6/37 (16.2)	1/19 (5.3)	5/18 (27.8)	0.09
Alb <40 g/L (%)	24/37 (64.9)	9/19 (47.4)	15/18 (83.3)	0.038
AST >40 U/L (%)	4/37 (10.8)	3/19 (15.8)	1/18 (5.6)	0.604
ALT >40 U/L (%)	2/37 (5.4)	1/19 (5.3)	1/18 (5.6)	1
TBL >17.1 mmol/L (%)	13/37(35.1)	5/19 (26.3)	8/18 (44.4)	0.313
D-dimer >0.5 mg/L (%)	7/37 (18.9)	4/19 (21.1)	3/18 (16.7)	1
Fib >4 g/L (%)	9/37 (24.3)	4/19 (21.1)	5/18 (27.8)	0.714
ESR ≥20 mm/h (%)	24/37 (64.9)	10/19 (52.6)	14/18 (77.8)	0.17
**Chest CT scan finding**				
Pneumonia (%)	32/37 (86.5)	16/19 (84.2)	16/18 (88.9)	1
Bilateral infiltration (%)	22 (68.8)	9 (56.2)	13 (81.2)	0.252
Unilateral infiltration (%)	10 (31.2)	7 (43.8)	3 (18.8)	

*PSI, pneumonia severity index; WBC, white blood cells; L, lymphocytes; SAA, serum amyloid A; CRP, C-reactive protein; PCT, procalcitonin; LDH, lactate dehydrogenase; Alb, albumin; AST, aspartate aminotransferase; ALT, alanine aminotransferase; TBL, total bilirubin; Fib, fibrinogen; ESR, erythrocyte sedimentation rate*.

**Table 2:** Disease severity classification among patients and complications and treatment measures instituted before release from quarantine.

**Characteristics**	**All patients (*n* = 37)**	**Duration from onset to release from quarantine**		
		**≤20-day (*n* = 19)**	**>20-day (*n* = 18)**	***P*-value**
Disease severity				0.738
Mild (%)	5 (13.5)	3 (15.8)	2 (11.1)	
Moderate (%)	30 (81.1)	15 (78.9)	15 (83.3)	
Severe (%)	1 (2.7)	0 (0)	1 (5.6)	
Critical (%)	1 (2.7)	1 (5.3)	0 (0)	
Complications				1
ARDS (%)	2 (5.4)	1 (5.3)	1 (5.6)	
**Treatment**				
Antibiotics	27 (73)	12 (63.2)	15 (83.3)	0.269
Intravenous antibiotics (%)	17/27 (63.0)	9/12 (75.0)	8/15 (53.3)	0.424
Oral antibiotics (%)	10/27 (37.0)	3/12 (25.0)	7/15 (46.7)	
Antifungal drugs	1/37 (2.7)	0/19 (0)	1/18 (5.6)	
Antiviral drugs (%)	37/37 (100)	19/19 (100)	18/18 (100)	0.17
Two (%)	25 (67.6)	15 (78.9)	10 (55.6)	
Three (%)	12 (32.4)	4 (21.1)	8 (44.4)	
Glucocorticoids (%)	8 (21.6)	4 (21.1)	4 (22.2)	1
Daily dose				0.131
40 mg (%)	6/8 (75)	4/4 (100)	2/4 (50.0)	
80 mg (%)	1/8 (12.5)	0/4 (0)	1/4 (25.0)	
120 mg (%)	1/8 (12.5)	0/4 (0)	1/4 (25.0)	
Albumin (%)	12 (32.4)	6 (31.6)	6 (33.3)	1
Immunoglobulin (%)	7 (18.9)	2 (10.5)	5 (27.8)	0.232
Thymosin (%)	24/37 (64.9)	9/19 (47.4)	15/18 (83.3)	0.038
Oxygen therapy (%)	15/37 (40.5)	7/19 (36.8)	8/18 (44.4)	0.743
Common (%)	13/15 (86.7)	6/7 (85.7)	7/8 (87.5)	1
HFNC (%)	2/15 (13.3)	1/7 (14.3)	1/8 (12.5)	-
PE (%)	1/37 (2.7)	0/19 (0)	1/18 (5.6)	0.486
TCM (%)	37/37 (100)	19/19 (100)	18/18 (100)	-

*HFNC, high-flow nasal cannula; PE, plasma exchange; TCM, traditional Chinese medicine*.

Patients in the >20-day group were older and had a longer duration from onset to hospitalization (*P* > 0.05). Regarding the clinical symptoms on admission, all of the seven asymptomatic patients belonged to the ≤20-day group. The rates of the symptoms of fatigue, sore throat, and shortness of breath were higher in the >20-day group (Table 1). These patients were more frequently treated with albumin and thymus peptide (Table 2) and had longer hospital stays (Table 3).

**Table 3:** Clinical outcomes of patients.

**Outcomes**	**All patients (*n* = 37)**	**Duration from onset to release from quarantine**		
		**≤20-day (*n* = 19)**	**>20-day (*n* = 18)**	***P*-value**
Hospitalization duration (d)	16.1 ± 6.2	12.4 ± 3.7	20.1 ± 5.8	0
Duration from onset to release from quarantine (d)	20.2 ± 6.6	15.1 ± 3.4	25.6 ± 4.4	0
**Blood findings at the time of release from quarantine**				
WBC <4.0 × 10^9^/L (%)	6/37 (16.2)	2/19 (10.5)	4/18 (22.2)	0.405
L <1.1 × 10^9^/L (%)	22/37 (59.5)	10/19 (52.6)	12/18 (66.7)	0.508
L ≤ 0.6 × 10^9^/L (%)	4/22 (18.2)	2/10 (20.0)	2/12 (16.7)	1
SAA>10 mg/L (%)	5/37 (13.5)	3/19 (15.8)	2/18 (11.1)	1

*WBC, white blood cells; L, lymphocytes; SAA, serum amyloid A*.

There was no significant improvement in granulocyte counts at the time of release from quarantine compared to the time of admission (Table 4).

**Table 4:** Granulocyte counts on admission and release from quarantine.

**Granulocyte counts**	**All patients (*****n*** **=** **37)**			**≤20-day (*****n*** **=** **19)**			**>20-day (*****n*** **=** **18)**		
	**Admission**	**Release**	***P*-value**	**Admission**	**Release**	***P*-value**	**Admission**	**Release**	***P*-value**
WBC (× 10^9^/L)	5.26 ± 2.15	5.02 ± 1.34	0.56	5.95 ± 2.38	5.33 ± 1.30	0.33	4.54 ± 1.64	4.67 ± 1.34	0.8
L (× 10^9^/L)	0.94 (0.73)	1.05 (0.58)	0.599	0.8 (0.76)	1 (0.6)	0.5	1.1 (0.74)	1.1 (0.31)	0.894
WBC <4.0 × 10^9^/(%)	10/37 (27.0)	6/37 (16.2)	0.398	4/19 (21.1)	2/19 (10.5)	0.66	6/18 (33.3)	4/18 (22.2)	0.715
L <1.1 × 10^9^/(%)	23/37 (62.2)	22/37 (59.5)	1	12/19 (63.2)	10/19 (52.6)	0.743	11/18 (61.1)	12/18 (66.7)	1
L ≤ 0.6 × 10^9^/(%)	10/23 (43.5)	4/22 (18.2)	0.108	6/12 (50.0)	2/10 (20.0)	0.204	4/11 (36.4)	2/12 (16.7)	0.371

*WBC, white blood cells; L, lymphocytes*.

## Discussion

Although the small number of cases included in this study affected the statistical analysis of some of the variables, many important characteristics were noted.

A majority of the 37 patients had mild and moderate disease, with only one severe case, and one critically ill case. All patients were finally released from quarantine without death, and the clinical outcome was significantly better than that observed in Hubei province (1, 5, 6, 12). The main reasons for this may be that the patients had relatively mild disease and that the availability of adequate medical facilities and personnel made the patients less likely to experience severe or critical conditions. After hospitalization, all patients were stratified according to PSI, which may be a better strategy to improve the outcome of COVID-19 patients, particularly in an outbreak when medical resources are relatively insufficient (13). Although the proportion of patients with chronic underlying diseases in this group was 21.6%, it did not seriously affect the outcome, since most of the patients were about 44 years old. According to the epidemiological histories, some of the patients in this study had been in direct contact with patients with confirmed disease. Some worked at the same workplace and did not meet the conceptual standard of close contact, which suggested the existence of a transmission route of COVID-19 via aerosol. Among these patients, asymptomatic patients constituted 18.9% of all patients. In some patients, a chest CT scan still revealed pneumonia and decreased white blood cell and lymphocyte counts. Among the first few symptoms recorded on admission, the rate of shortness of breath and gastrointestinal symptoms was high, which did not exclude the influence of psychological factors. In addition, the proportion of patients with fever was not high, and the rate of fever was low. These characteristics were different from those of other highly infectious viral respiratory infections (14, 15). Among the variables assessed on routine blood biochemistry tests on admission, the most common was a reduction in the lymphocyte count. In nearly half of these patients, the lymphocyte count was ≤0.6 × 10^9^/L. White blood cell counts were mostly normal; less than a third of patients had lower white blood cell counts. Other abnormalities with relatively high proportions were the levels of ESR, albumin, SAA, and CRP; however, most of the changes were slight and less specific. Thus, a decrease in lymphocyte count may be the most important feature in routine biochemical tests (1, 6). Changes in chest CT scans observed in this group were similar to those observed in other COVID-19 studies and were significantly different from the characteristics of H1N1 pneumonia (16).

Treatment measures instituted among the patients in this group were mainly performed in accordance with the protocol (8, 9). Although the patients mainly had mild and moderate conditions, their treatment was complicated due to the particularity of the epidemic (17, 18). Two-thirds of the patients were given antibiotics, although there was not sufficient evidence of bacterial infection. Although no specific antiviral drugs were recommended, the patients in this group were given antiviral drugs routinely; two-thirds were given two antiviral drugs, and one-third were given three antiviral drugs. The effects of thymosin, glucocorticoid, albumin, and immunoglobulin on COVID-19 need to be investigated further, particularly in patients with mild and moderate disease. Many studies have demonstrated the important role of TCM in inhibiting coronavirus (19–21).

Although the patients in this study mainly had mild and moderate disease, there were significant differences in the duration from symptom onset to release from quarantine. The most important basis for release from quarantine is the persistence of negative nucleic acid test results. Thus, the duration from symptom onset to release from quarantine reflects the time it takes for the virus to be released from the respiratory tract of the patient. The average time from the onset of symptoms to release from quarantine was 20 days. Patients could only be released from quarantine after three consecutive negative nucleic acid test results, tests could be performed at 24-h intervals, and the incubation period reported in previous literature was considered (1); thus, the average duration of virus release in this study should be similar to that reported by Zhou et al. (22).

Some items in the ≤20-day and >20-day groups were significantly different, which may explain why the patients could not be released from quarantine for a long time. Patients in the >20-day group were older, and the time from onset to admission was longer, suggesting that although there was no specific antiviral drug for COVID-19, systematic supportive treatment administered after admission could improve outcomes, even among patients with mild and moderate disease. There were no differences in PSI score, underlying chronic disease, or epidemiological history on admission between the two groups, possibly due to the small number of cases or mild illness. Regarding symptoms reported on admission, seven asymptomatic patients screened recovered quickly, which may be related to the viral load and individual differences. Among symptomatic patients, fatigue and pharyngeal pain were more obvious among patients in the >20-day group, for unknown reasons. In terms of routine blood biochemical examination and pulmonary imaging, although the proportions of individual abnormal indicators in the >20-day group were higher than those in the ≤20-day group, the number of samples was not large enough to yield sufficient clinical significance. There was no significant difference in the number of patients between the two groups, which was related to the fact that almost all the patients had mild and moderate diseases, while one critically ill patient was quickly released from quarantine. These clinical results may suggest that there is a cross relationship between sustained positive test results for nucleic acid to SARS-CoV-2 observed in respiratory tract specimens and the severity of the disease (1, 22, 23). However, it was not a linear relationship, and the reasons for the sustained positive nucleic acid test results are complex. The >20-day group received more drugs, which may be related to eagerness to ensure that negative nucleic acid test results were obtained.

There was no significant improvement in the white blood cell and lymphocyte counts at the time of release from quarantine and at the time of admission in either the ≤20 or >20-day groups. The reasons need to be studied further.

This study had certain limitations. First, the number of cases in this study was not large; it had obvious regional characteristics, and the majority of patients had mild and moderate diseases, which cannot represent the characteristics of a large number of patients in a large geographical range. The study was also not representative of patients with severe and critical conditions. Nevertheless, this study can still provide a reference and help in the prevention and control of COVID-19 in other comparable smaller-sized outbreaks.

## Conclusions

Most cases of COVID-19 recorded in Liaocheng city were mild and moderate. The main source of infection was exposure to a patient with confirmed disease or to the workplace of a patient with confirmed disease. The main clinical symptoms were cough, fever, and fatigue; however, shortness of breath, sore throat, and gastrointestinal symptoms were also common. A chest CT scan showing features of pneumonia and a reduced lymphocyte count were the most important adjunctive examination findings. The duration between symptom onset and release from quarantine was related to age, the length of time from onset to admission, and the presence or absence of symptoms and was not related to the mildness or normality of the type. There was no significant improvement in white blood cell and lymphocyte counts at the time of release from quarantine compared to the time of admission.

## Data Availability Statement

With the permission of the corresponding author, we can provide participant data for further statistical analysis.

## Ethics Statement

The study was conducted in accordance with the principles of the Declaration of Helsinki, and the study protocol was approved by the Ethics Committee of Liaocheng People's Hospital. The need for informed consent was waived on account of the retrospective nature of the study.

## Author Contributions

ST and TW had full access to all of the data in the study and take responsibility for the integrity of the data and the accuracy of the data analysis. ST and ZC contributed to study design and writing. YW, MW, WZ, GZ, TX, and JX contributed to acquisition, analysis, or interpretation of data. TW, XZ, and HT performed critical revision of the manuscript for important intellectual content. JC, JH, and KN contributed to study supervision.

## Conflict of Interest

The authors declare that the research was conducted in the absence of any commercial or financial relationships that could be construed as a potential conflict of interest.
